# Development, Optimization, and Stability Study of a Yataprasen Film-Forming Spray for Musculoskeletal Pain Management

**DOI:** 10.3390/gels11010064

**Published:** 2025-01-15

**Authors:** Jaenjira Angsusing, Weerasak Samee, Sarin Tadtong, Supachoke Mangmool, Siriporn Okonogi, Nopparut Toolmal, Chuda Chittasupho

**Affiliations:** 1 Ph.D. Degree Program in Pharmacy, Faculty of Pharmacy, Chiang Mai University, CMU Presidential Scholarship, Chiang Mai 50200, Thailand; 2 Department of Thai Traditional and Alternative Medicine, Ministry of Public Health, Bangkok 10100, Thailand; 3 Department of Pharmaceutical Chemistry, Faculty of Pharmacy, Srinakharinwirot University, Ongkharak, Nakhon Nayok 26120, Thailand; 4 Department of Pharmacognosy, Faculty of Pharmacy, Srinakharinwirot University, Ongkharak, Nakhon Nayok 26120, Thailand; 5 Department of Pharmaceutical Care, Faculty of Pharmacy, Chiang Mai University, Chiang Mai 50200, Thailand; 6 Department of Pharmaceutical Sciences, Faculty of Pharmacy, Chiang Mai University, Mueang, Chiang Mai 50200, Thailand; 7 Center of Excellence in Pharmaceutical Nanotechnology, Faculty of Pharmacy, Chiang Mai University, Chiang Mai 50200, Thailand

**Keywords:** film-forming spray, natural products, stability study, Yataprasen

## Abstract

Yataprasen (YTPS) remedy ethanolic spray, one of the National Thai Traditional Medicine Formulary, is extensively employed in Thai traditional healthcare to manage musculoskeletal pain and inflammation. Despite its widespread use, the quality and stability of the YTPS formulation, critical to its efficacy, safety, and patient adherence, have not been comprehensively studied. This research developed and optimized a film-forming spray (FFS) formulation of YTPS ethanolic extract and conducted a 6-month stability evaluation. The FFS shares similarities with gel formulations, particularly in its ability to form a cohesive, semi-solid film upon application, enhancing localized drug delivery and prolonged contact time. Key physicochemical properties, including density (0.8450–0.9086 g/cm^3^), pH (4.72–4.95), spray angle (55.58–60.10°), evaporation time (1.04–1.27 min), and theoretical film thickness (7.72–13.97 µm), were analyzed across varying storage conditions. Active components β-amyrin and stigmasterol demonstrated retention rates of 96.78% and 68.22%, respectively, under refrigerated conditions, with degradation rates accelerating at higher temperatures. Significant variations in density, spray angle, film thickness, and stigmasterol concentration were observed. Additionally, the RP-HPLC method was validated for the accurate and precise quantification of the bioactive compounds such as β-amyrin and stigmasterol, demonstrating excellent linearity within a 10–100 µg/mL range for both compounds with excellent linearity R^2^ > 0.999. The results confirmed that YTPS-FFS exhibits good stability and that the validated HPLC method is reliable for routine quality control. These findings supported the potential of YTPS-FFS formulation as a standardized and effective dosage form for managing musculoskeletal conditions, advancing its role in modernized traditional medicine.

## 1. Introduction

Yataprasen (YTPS) remedy formulary, recognized as the 58th entry in a traditional Thai medical compendium, is a nationally acknowledged component of Thai traditional medicine. This formulary contains a blend of 13 medicinal herbs historically used to alleviate symptoms associated with musculoskeletal discomfort, including pain from strain, cramps, and joint pain. Traditionally, YTPS is prepared in powdered form and applied using a vehicle of 40% ethanol or naturally fermented vinegar to enhance absorption and efficacy [1].

Chronic musculoskeletal pain is the leading cause of disability. It encompasses acute or chronic discomfort in bones, muscles, ligaments, tendons, and nerves, significantly diminishing quality of life and imposing heavy burdens on healthcare systems and disability insurance. Common forms include low back pain, neck pain, osteoarthritis (OA), and rheumatoid arthritis, although it also includes conditions such as sprains and fractures. Risk increases with age, but musculoskeletal pain affects individuals across all age groups, often leading to persistent or recurrent symptoms with physical, psychological, and socioeconomic consequences. The clinical management of pain generally relies on oral nonsteroidal anti-inflammatory drugs (NSAIDs) [2]. However, prolonged use of NSAIDs is associated with a range of adverse effects, including cardiovascular, renal, gastrointestinal, and hematological complications. Enhancing the therapeutic safety profile of pain management, possibly through alternative delivery methods such as topical NSAIDs, is thus critical to optimizing long-term clinical outcomes in pain management without compromising symptom control [3]. Recent studies have shown that topical NSAIDs have demonstrated comparable efficacy to oral NSAIDs in the management of OA, effectively reducing pain and enhancing physical function among OA patients. Both administration routes have shown similar therapeutic outcomes, relieving OA-associated symptoms. Topical NSAIDs may potentially offer a safer alternative by minimizing systemic exposure [4].

The YTPS ethanolic extract was analyzed from our previous study to identify its primary compounds, β-amyrin and stigmasterol. The extract exhibited significant in vitro anti-inflammatory properties at non-toxic concentrations, effectively suppressing the production of key pro-inflammatory cytokines, including IL-1β, IL-6, and TNF-α [5,6]. Furthermore, it demonstrated a dose-dependent reduction in nitric oxide (NO) production [5,6], a critical mediator of inflammatory responses. Notably, the extract showed no cytotoxic effects on renal cells, underscoring its safety profile [5]. These findings highlight the therapeutic potential of the YTPS ethanolic extract as a promising candidate for developing topical anti-inflammatory treatments. Its efficacy in modulating inflammatory pathways suggests its application in managing pain and other inflammatory conditions, positioning it as a viable alternative to conventional therapies.

Transdermal treatments are designed to reach systemic therapeutic levels of active pharmaceutical ingredients comparable to those achieved with oral administration, employing percutaneous absorption for drug delivery. Although these treatments typically exhibit a delayed onset of action, they frequently provide sustained therapeutic effects due to gradual drug release over time [7]. In contrast, topical agents are intended for localized cutaneous delivery, focusing their therapeutic effects directly on the application site. By targeting the underlying soft tissues and nerves at the application area, topical agents minimize systemic drug absorption. Consequently, serum concentrations remain low, reducing the likelihood of systemic adverse events and minimizing the potential for drug interactions, thus offering a favorable safety profile for localized management [8].

To enhance therapeutic efficacy and optimize pharmacokinetic profiles, topical drugs are typically formulated in delivery systems such as patches, gels, lotions, creams, ointments, or sprays. Recent innovations have led to the development of film-forming sprays (FFS), which have gained traction in the pharmaceutical field. Unlike patches, FFS is nearly invisible, providing esthetic appeal while maintaining non-sticky, non-greasy skin feels. They facilitate precise dose adjustment, sustained release, and efficient topical or transdermal delivery. Additionally, their ability to adapt to localized or systemic drug delivery needs enhances therapeutic flexibility, making FFS a versatile and patient-friendly option in topical drug administration [9]. The FFS formulation exhibits notable similarities to gel formulations, particularly in its ability to transform into a cohesive, semi-solid film upon application. This film formation not only facilitates effective adherence to the application site but also enhances localized drug delivery by creating a controlled-release matrix. Additionally, the semi-solid film prolongs contact time with the skin or mucosal surface, thereby improving the retention of active pharmaceutical ingredients and optimizing therapeutic efficacy. Such properties align the FFS formulation with the functional characteristics of gels, positioning it as an innovative alternative for targeted and sustained drug delivery applications. FFS consists of active agents, permeation enhancers, and polymers in organic solvents, forming a thin, non-sticky film that enhances drug contact time, permeability, and controlled release. This formulation minimizes crystallization, ensuring a more consistent therapeutic effect than conventional topical preparations [8,9].

Despite its widespread use, the quality and stability of the YTPS formulary, which directly impacts its therapeutic efficacy, potential adverse effects, and patient adherence, still need to be studied more. Unlike modern pharmaceuticals, which have undergone extensive dosage form development to optimize therapeutic outcomes, ensure uniformity, enhance efficacy, minimize side effects, and improve patient compliance, traditional herbal medicines often lack such advancements in formulation. Consequently, the dosage forms of many traditional remedies remain rudimentary and less refined. This gap underscores the critical need for rigorous research to modernize its formulation, standardize its quality, and substantiate its safety and therapeutic claims for broader and safer clinical applications. The primary objective of this study was to formulate and evaluate a FFS for the YTPS formulation. The investigation included an evaluation of the physicochemical properties along with the chemical composition of the YTPS film-forming spray (YTPS-FFS) following a 6-month stability study. This analysis yielded valuable insights into the formulation’s potential for therapeutic application. Such an approach highlights the significance of innovative formulation techniques in optimizing drug delivery systems, ultimately contributing to improved clinical outcomes.

## 2. Results and Discussion

### 2.1. Pre-Formulation of FFS Base

The FFS system offers easy application, uniform coverage, and prolonged localized drug delivery, making it ideal for topical treatments [8]. Its ability to form a cohesive and durable film enhances its practicality and effectiveness. Pre-formulation studies are critical for optimizing key physical properties like solubility, viscosity, sprayability, and drying rate [9]. Evaluating factors like appearance and film characteristics ensures the development of formulations that achieve consistent and reliable film formation. This study examined 10 formulations with varying excipient compositions to identify their influence on these key properties. This systematic approach highlighted the importance of balancing formulation dynamics for effective performance and applications.

The study of physical properties of the FFS base focused on critical attributes, including appearance, viscosity, sprayability, drying rate, and film characteristics (Table 1). Among the 10 formulations, the majority (formulations 1, 4–10) demonstrated a clear appearance, suggesting adequate solubility and uniform dispersion of excipients [10]. However, formulations 2 and 3 exhibited turbid and white–turbid appearances, respectively. This turbidity is likely due to incomplete dissolution or incompatibility among the ingredients, which can lead to phase separation or precipitation [10].

Viscosity is a crucial parameter that affects the sprayability and uniform distribution of the formulation [11]. High viscosity, as observed in formulations 2 and 3, hindered the sprayability, leading to inadequate atomization and poor coverage on the target surface. Conversely, low-viscosity formulations, such as 6–9, exhibited good sprayability, highlighting the importance of maintaining an optimal viscosity range for ease of application. Medium-high viscosity, as seen in formulation 1, resulted in poor sprayability, indicating that even moderately viscous formulations may struggle to achieve efficient aerosolization. The findings suggest that low viscosity is preferable for FFS products, as it facilitates uniform droplet formation and reduces nozzle clogging.

Sprayability, a critical attribute for FFS formulations, was assessed based on the ease of expelling the solution from the spray bottle and the uniformity of the spray pattern. Most formulations (4–10) demonstrated good sprayability, particularly those with low viscosity. This consistency indicates that low-viscosity formulations are more likely to achieve optimal spray characteristics. Formulation 1 exhibited poor sprayability, which can be attributed to its medium-high viscosity. Formulations 2 and 3 were not assessed for sprayability due to their turbid nature and high viscosity, which rendered them unsuitable for practical application.

The drying rate of the formulations was another critical factor influencing film formation [12]. A slow drying rate, as observed in formulations 4, 5, and 6, prevented the formation of effective films, which might be due to prolonged solvent evaporation times that disrupted the uniformity of the deposited layer. Formulations 7, 8, and 10 had medium drying rates, which improved the potential for film formation but did not consistently result in completely formed films. Formulation 9, with a medium-to-high drying rate, achieved the best balance, allowing sufficient time for the solution to spread evenly before drying while preventing disruptions caused by excessively prolonged evaporation.

The ultimate goal of an FFS formulation is to form a cohesive, uniform film upon application [8]. Among the 10 formulations, only formulation 9 successfully achieved complete film formation. Other formulations faced challenges in film formation. Formulations 1–6 failed to form any film, despite varying levels of sprayability and drying rates. The lack of film-forming agents or inadequate plasticizer content may have contributed to these failures. The formed films in formulations 7 and 8 were incomplete, suggesting that while the drying rates were sufficient, the formulations lacked the necessary cohesion or mechanical strength for complete film development. In formulation 10, while a film was formed, it appeared white and turbid, signifying physical incompatibilities or phase separation during the drying process.

Formulation 9 was identified as the optimal candidate for further development, exhibiting a combination of desirable characteristics, including a clear visual appearance, low viscosity, excellent sprayability, a medium-to-high drying rate, and the ability to form a complete and uniform film. These properties contributed to enhanced performance by enabling uniform dispersion, controlled solvent evaporation, and effective polymer interactions, which collectively resulted in the formation of a durable and cohesive film. The superior attributes of formulation 9 underscore its suitability for practical applications and emphasize the importance of these critical parameters in the successful development of FFS formulations.

Polyvinylpyrrolidone K30 (PVP K30) is a versatile polymer commonly employed in pharmaceutical and cosmetic formulations due to its solubility, biocompatibility, and multifunctional properties. As a gelling agent, PVP K30 enhanced the viscosity of formulations, stabilizing semi-solid systems and forming gel-like matrices when combined with suitable excipients. These properties are particularly advantageous in creating cohesive systems that facilitate controlled drug delivery [13].

PVP K30 also acts as an effective film-forming polymer, bridging the functionalities of gels and FFS. In FFS, PVP K30 transitions from a liquid or semi-solid state to a cohesive, semi-solid film upon solvent evaporation, mimicking the extended surface coverage and adhesion properties typical of gels [14]. The formation of a solid film involves several processes, including diffusion, gelation, and glass transition. These processes are interdependent and dictate the final structure of the film [15,16]. This film enhanced localized drug delivery, prolonged the contact time of active ingredients, and offered controlled-release characteristics similar to gel systems.

### 2.2. Physical Characterization and Stability of the YTPS-FFS

The physical characterization and stability assessment of the YTPS-FFS formulation are comprehensively presented in Table 2. These data provided critical insights into the formulation’s physicochemical properties, such as appearance, density, pH values, stickiness, water washability, evaporation time, spray angle, weight delivered after every actuation, weight, and theoretical film thickness, as well as its stability under various storage conditions over time. In addition, the viscosity of the formulation was found to be 0.015 ± 0.000 Pa·s, indicating acceptable fluidity suitable for spray application. The evaluation of these parameters is essential for determining the robustness and suitability of the YTPS-FFS for practical applications. Table 2 also highlights the stability performance of the formulation, including its resistance to degradation of active compounds. This analysis is particularly significant for predicting the long-term viability and shelf-life of the YTPS-FFS, ensuring that its therapeutic or functional properties remain intact under intended storage conditions. These findings not only validate the formulation’s stability but also provide a foundation for future optimization and potential scale-up for industrial or pharmaceutical use.

#### 2.2.1. Appearance

The appearance of the film-forming spray is a key parameter in evaluating its quality and user acceptance. The YTPS-FFS was visually assessed and found to exhibit a clear green color and homogeneous appearance without phase separation (Supplementary Figure S1). There were some precipitated visible particles in the bottom of the spray bottle. This observation was unchanged from the start to the last time point of the stability study. A stable and esthetically acceptable appearance is crucial for maintaining consumer confidence and ensuring product performance.

#### 2.2.2. Density

Across all temperatures, the density values fluctuated slightly but remained within acceptable ranges. At 4 °C, the density started at 0.8896 g/cm^3^ and showed a slight dip to 0.8450 g/cm^3^ at one month, followed by stabilization around 0.9086 g/cm^3^ and 0.8471 g/cm^3^ at three and six months, respectively. A similar trend was observed at 30 °C, where the density ranged from 0.8831 g/cm^3^ initially to 0.8475 g/cm^3^ by six months. At 40 °C, density values were comparable but showed more variation, starting at 0.8885 g/cm^3^ and dropping to 0.8397 g/cm^3^ at six months. These findings indicate that the formulation maintained its structural integrity across all conditions, although higher temperatures caused slightly greater fluctuations.

#### 2.2.3. pH Values

Since human skin pH varies between 4.1 and 5.8, the topical products should be acidified to achieve a pH within the range of 4 to 6 [17]. This pH range is important for enhancing product stability and compatibility with the skin, thereby optimizing therapeutic efficacy while minimizing the risk of irritation [18]. The pH of a formulation influences its stability, compatibility, and user comfort. The pH values of the YTPS-FFS formulation were consistently within the range of 4.72 to 4.95. There were no statistically significant differences observed in pH levels throughout the stability study period when compared to the freshly prepared YTPS-FFS. At 4 °C, the pH remained stable, starting at 4.88 and ending at 4.89 after six months, showing minimal variation. At 30 °C, the pH values ranged from 4.93 initially to 4.79 at six months, demonstrating a gradual decline. At 40 °C, the pH showed more significant variation, with values dropping from 4.72 initially to 4.73 after six months. The consistent pH at lower temperatures suggests better chemical stability, while higher temperatures likely accelerated degradation processes, causing slight acidification.

#### 2.2.4. Stickiness

The stickiness of the formulation was consistently reported as “low” across all time points and temperatures, indicating a favorable tactile profile. Similarly, the washability remained rated as “Good (less than 1 min)”, reflecting excellent performance in this aspect. These results suggest that the formulation’s usability and ease of removal were not adversely affected by storage conditions or time.

#### 2.2.5. Water Washability

Topical products are expected to be easily removed with water [18]. The YTPS-FFS formulation demonstrated the ability to be consistently rinsed off with water in less than 1 min from the beginning to the conclusion of the stability testing period. This characteristic was maintained throughout the entire evaluation, indicating that the formulation preserved its washability under the experimental conditions.

#### 2.2.6. Evaporation Time

Evaporation time is critical for determining drying characteristics. At 4 °C, the average evaporation time was consistent, ranging from 1.18 min initially to 1.13 min at six months. At 30 °C, evaporation time peaked slightly at 1.27 min at one month but stabilized around 1.14 min by six months. At 40 °C, the evaporation time showed more fluctuation, starting at 1.11 min, dipping to 1.04 min at three months, and rising to 1.17 min by six months. These findings suggest that lower temperatures helped maintain uniform evaporation times, whereas higher temperatures led to slight variability. The evaporation time for an FFS formulation is well within the acceptable threshold, being under 4 min. This characteristic is crucial for ensuring patient convenience and compliance, as rapid evaporation facilitated quick drying of the spray on the skin, forming a protective film without prolonged waiting. Such efficiency not only enhances the user experience but also minimizes the risk of smudging or transferring the formulation before the film is fully formed, thereby contributing to the reliability and effectiveness of the topical drug delivery system [19].

#### 2.2.7. Spray Angle

Spray angle measurements provided insights into the consistency of the dispensing mechanism. At 4 °C, the spray angle remained stable, averaging around 58.41° to 59.18° across six months. At 30 °C, the spray angle ranged from 58.69° initially to 57.65° at six months. At 40 °C, more variability was observed, with the spray angle decreasing from 60.10° initially to 55.58° at six months. These variations at higher temperatures might indicate slight mechanical changes in the formulation’s dispensing properties under thermal stress. A spray angle of less than 85° is considered optimal for spray formulations, as it ensures efficient actuation while maximizing the area of coverage. This angle facilitated the uniform application of the formulation, enhancing its usability and effectiveness. By maintaining a controlled spray pattern, the formulation achieved consistent distribution across the target area, which is particularly critical for therapeutic applications requiring precise and even coverage [20].

#### 2.2.8. Weight Delivered After Every Actuation

The weight delivered after each actuation of the FFS was measured to ensure the consistency and reliability of the formulation’s application. This parameter is critical for determining the dosage and effectiveness of the spray in delivering therapeutic compounds to the intended applications. The weight delivered per actuation was consistent across 4 °C and 30 °C, with only minor changes. At 4 °C, it ranged from 0.1274 g initially to 0.1193 g at six months, while at 30 °C, it ranged from 0.1434 g initially to 0.1279 g. At 40 °C, the weight varied more significantly, starting at 0.1227 g and dropping to 0.0957 g by six months. These reductions at higher temperatures suggest possible evaporation or changes in the formulation’s rheological properties. The weight of the YTPS-FFS delivered per actuation was found to align closely with the findings of Deshmukh [21], including Rajab [22] and Bakshi [23], who utilized a combination of poloxamer and carbomer as film-forming agents. This consistency underscores the potential reliability and uniformity of YTPS-FFS in delivering precise doses per actuation.

#### 2.2.9. Weight

Weight loss is indicative of evaporation or degradation of volatile components. At 4 °C, weight loss was negligible, with a total reduction of 0.15% *v/v* over six months. At 30 °C, weight loss was slightly higher, totaling 0.26 %*v*/*v*. At 40 °C, a significant weight loss of 2.78 %*v*/*v* was observed, with the most substantial decrease at 6 months. This pattern highlights the impact of higher temperatures on the formulation’s stability, likely due to increased volatilization rates. The observed weight loss in the YTPS-FFS can be attributed primarily to the presence of ethanol, a key component of the formulation. Ethanol’s low molecular weight and high volatility significantly contribute to its rapid evaporation during and after stability studies. Furthermore, the substantial proportion of ethanol utilized in the formulation amplifies this evaporation effect, ultimately resulting in the observed reduction in weight over time [24]. This characteristic of ethanol plays a crucial role in the rapid drying and film formation properties of YTPS-FFS, enhancing its overall functionality as a topical delivery system.

#### 2.2.10. Theoretical Film Thickness

The thickness of the film formed after spraying was approximately between 0.007 and 0.015 cm, or between 7.48 and 15.54 µm. Over 6 months under accelerated storage conditions, a significant reduction in film thickness was observed. The film thickness decreased from 13.30 ± 0.29 microns to 7.73 ± 0.34 µm. Theoretical film thickness reflects the formulation’s ability to form a consistent coating. At 4 °C, film thickness remained stable, averaging 12.05 µm initially and 11.91 µm by six months. At 30 °C, the thickness ranged from 13.97 µm initially to 11.97 µm at six months, showing a slight decline. At 40 °C, film thickness was initially 13.30 µm but dropped to 7.72 µm at six months, indicating a significant reduction. These findings suggest that higher temperatures negatively affected film formation, likely due to changes in the formulation’s viscosity or evaporation dynamics.

### 2.3. Validation Results for HPLC Method

#### 2.3.1. Specificity

**Figure 1:**
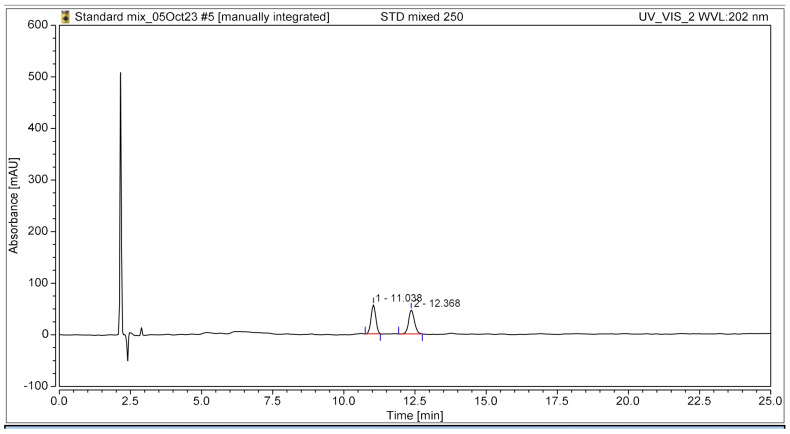
HPLC chromatogram of the YTPS-FFS spiked with β-amyrin and stigmasterol at 202 nm UV detection.

The HPLC chromatogram clearly illustrated the complete separation of the two bioactive compounds, β-amyrin and stigmasterol, in the YTPS-FFS formulation, without the presence of interfering peaks, as shown in Figure 1. The retention times were recorded at 11.04 min for β-amyrin and 12.37 min for stigmasterol, respectively.

#### 2.3.2. Linearity and Range

Linear regression analysis demonstrated a favorable linear relationship between the absorbance of β-amyrin and stigmasterol, and the concentrations ranged from 10 to 100 µg/mL, as illustrated in Supplementary Figure S2. The calibration curve, fitted using the least squares method, yielded the regression equation y = 0.2033x − 0.3596 and y = 0.1996x + 0.0874 with a good correlation coefficient of 0.9999 and 0.9998 for β-amyrin and stigmasterol, correspondingly.

#### 2.3.3. Accuracy

The accuracy was determined by calculating the %recovery of β-amyrin and stigmasterol at three concentration levels (50%, 100%, and 150%) spiked into a pre-analyzed YTPS-FFS solution. The difference between the theoretical concentration and the experimentally determined concentration in the spiked samples was expressed as %recovery. The results indicated that the mean recovery values for β-amyrin and stigmasterol ranged from 104% to 110% and 99% to 100%, respectively, as detailed in Table 3. Generally, for unregulated products, an acceptable recovery range is between 90% and 110%.

#### 2.3.4. Precision

The results demonstrated that the %RSD for both intra- and inter-day precision at all tested concentrations was below 2%, falling within the adequate criteria as demonstrated in Table 4. These findings confirmed that the method offered an acceptable level of precision for accurately determining β-amyrin and stigmasterol quantification on the YTPS-FFS sample formulation.

#### 2.3.5. Sensitivity

The limits of detection (LOD) for β-amyrin and stigmasterol in the YTPS-FFS formulation were 0.3798 µg/mL and 0.6471 µg/mL, respectively. The limits of quantification (LOQ) for β-amyrin and stigmasterol in the YTPS-FFS formulation were 1.2660 µg/mL and 2.1571 µg/mL, respectively.

#### 2.3.6. Robustness

The results indicated that minor adjustments in 2% of mobile phase composition and 13% of flow rate did not produce any significant changes, as shown in Table 5.

#### 2.3.7. System Suitability

To assess system suitability, crucial chromatographic parameters such as retention time, resolution, theoretical plate count, separation factor, retention factor, asymmetry, and tailing factor were evaluated for active compounds. A standard mixture of 60 µg/mL was injected to determine these system suitability parameters. The resolution between the two peaks was found to be 3.09 ± 0.25, indicating well-separated peaks. Additionally, the values for peak resolution, theoretical plates, separation factor, retention factor, asymmetry, and, tailing factors were within adequate limits, as displayed in Table 6.

Chemical standardization of polyherbal formulations is a critical process to ensure their quality, efficacy, and safety [25]. HPLC is widely utilized for the quantification of bioactive markers in these formulations due to its precision, sensitivity, and reproducibility [26]. The analysis of drug content in the YTPS-FFS was conducted using the HPLC technique. The RP-HPLC analytical method was developed to simultaneously quantify the bioactive compounds in the YTPS ethanolic extract, β-amyrin and stigmasterol, found in *Putranjiva roxburghii* Wall, which are major components in the YTPS formulary [27]. The retention times of both compounds demonstrated the specificity of the developed method at a detection wavelength of 202 nm, consistent with the findings of Sharma et al. [28]. However, notable differences were observed in the mobile phase composition and flow rate. Specifically, a higher proportion of the non-polar solvent acetonitrile was utilized, along with adjustments to the flow rate, resulting in increased retention times in the current method.

Additionally, the LOD and LOQ were significantly lower than those reported by Sharma et al., enhancing the method’s sensitivity. The linearity range of 10–100 µg/mL in the current study markedly outperformed the 150 µg/mL lower limit reported by Sharma et al. [28]. Robustness testing was conducted in a comparable manner, evaluating variations in wavelength, flow rate, and mobile phase ratios. However, the parameters for wavelength and flow rate were subjected to more rigorous modifications in Sharma et al.’s [24] study compared to the present work. The purpose method exhibits a simple, accurate, precise, and less time-consuming routine analysis of the YTPS-FFS.

#### 2.3.8. Kinetic Degradation of β-Amyrin and Stigmasterol in the YTPS-FFS During Storage

**Figure 2:**
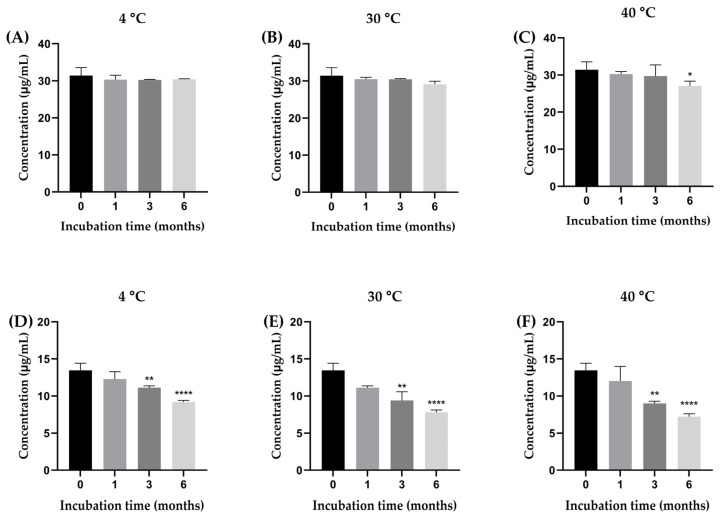
The concentration of β-amyrin (**A**–**C**) and stigmasterol (**D**–**F**) in the YTPS-FFS after 6 months of stability study at three different storage conditions (4, 30, and 40 °C). Results are expressed as the mean ± SD based on analysis of triplicate samples (*n* = 3). The data were analyzed by GraphPad Prism version 9.5.0 with one-way ANOVA and Dunnett’s multiple comparisons test (statistical significance denoted by * *p* < 0.05, ** *p* < 0.01 and **** *p* < 0.0001 compared with initial concentration).

**Figure 3:**
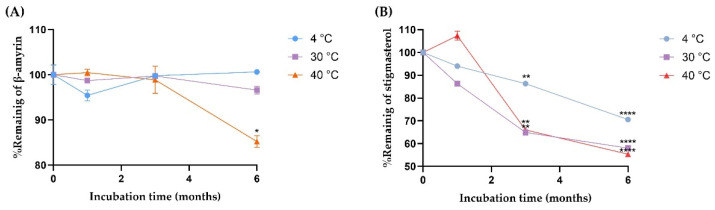
The degradation kinetics of (**A**) β-amyrin and (**B**) stigmasterol at three different storage conditions (4, 30, and 40 °C) Results are expressed as the mean ± SD based on analysis of triplicate samples (*n* = 3). The data were analyzed by GraphPad Prism version 9.5.0 with one-way ANOVA and Dunnett’s multiple comparisons test (statistical significance denoted by * *p* < 0.05, ** *p* < 0.01, and **** *p* < 0.0001 compared with % remaining at initial concentration).

The stability of β-amyrin and stigmasterol in the YTPS-FFS formulation was systematically evaluated over a 6-month period under storage conditions of 4 °C, 30 °C, and 40 °C by quantifying with HPLC analysis, as illustrated in Figure 2. As presented in Figure 3, the percentage of β-amyrin retained after 6 months exhibited a gradual decline across all storage temperatures. The remaining percentages were 96.78 ± 0.16% at 4 °C, 92.59 ± 0.85% at 30 °C, and 86.07 ± 1.30% at 40 °C. Notably, the degradation pattern adhered to a zero-order kinetic model, as demonstrated by the linear relationship between β-amyrin concentration and storage time, with high correlation coefficients (r^2^ = 0.3161, 0.9031, and 0.9603 at 4 °C, 30 °C, and 40 °C, respectively). The rate constants for degradation were calculated as 0.39% per month at 4 °C, 0.91% per month at 30 °C, and 1.71% per month at 40 °C, underscoring the temperature dependence of the degradation process. To further assess the impact of storage temperature, the shelf-life (t_90_), defined as the time required for the retention of 90% of the initial β-amyrin concentration, was estimated. The calculated t_90_ values were approximately 18.7 months at 4 °C, 8.1 months at 30 °C, and 4.3 months at 40 °C.

Stigmasterol exhibited a consistent decline in stability across all tested storage temperatures, with remaining percentages after six months recorded as 68.22 ± 0.23% at 4 °C, 58.00 ± 0.32% at 30 °C, and 53.71 ± 0.37% at 40 °C. This degradation behavior followed a second-order kinetic model, as evidenced by the strong linear correlation between the inverse of stigmasterol concentration and storage time. The corresponding r^2^ was notably high, at 0.9939, 0.9784, and 0.9904 for storage at 4 °C, 30 °C, and 40 °C, respectively, confirming the reliability of the second-order model in describing stigmasterol’s degradation dynamics. The rate constants for stigmasterol degradation were calculated as 1.18% per month at 4 °C, 1.32% per month at 30 °C, and 1.35% per month at 40 °C. Furthermore, the calculated shelf-life (t_90_) was determined to be approximately 1.42 months at 4 °C, 0.91 months at 30 °C, and 0.77 months at 40 °C.

Drug content analysis focused on the active compounds, β-amyrin and stigmasterol [7]. At 4 °C, β-amyrin content showed minor fluctuations, ranging from 30.19 µg/mL to 31.12 µg/mL, demonstrating excellent stability under cooler conditions. In contrast, stigmasterol content declined steadily, from 13.26 µg/mL at baseline to 7.50 µg/mL at six months, suggesting a moderate susceptibility to degradation at high temperatures. At 30 °C, β-amyrin exhibited more variability, peaking at 30.92 µg/mL at three months before dropping to 26.87 µg/mL by six months.

Stigmasterol experienced a more pronounced decline, falling from 13.26 µg/mL to 7.68 µg/mL within the same period. These results highlight that moderate temperatures accelerate degradation, particularly for stigmasterol. At 40 °C, both β-amyrin and stigmasterol showed significant instability. β-Amyrin dropped to 22.96 µg/mL at six months, while stigmasterol declined sharply to 7.60 µg/mL.

The stability study conducted using HPLC analysis quantified β-amyrin at approximately 30 µg/mL within the YTPS-FFS formulation. This concentration aligns well with effective doses reported in prior in vitro studies, where β-amyrin exhibited potent anti-inflammatory activity at concentrations ranging from 10 to 50 µg/mL [29,30]. Additionally, stigmasterol was detected at levels exceeding 5 µg/mL in the YTPS-FFS. Research by Sampath, S. J. P., et al. demonstrated that stigmasterol exerts significant anti-inflammatory effects in vitro at concentrations as low as 10 µg/mL [31]. Collectively, the concentrations of β-amyrin and stigmasterol in the YTPS-FFS fall within the established therapeutic ranges for anti-inflammatory efficacy. Following the recommended application directions, these levels are expected to provide sufficient local delivery of β-amyrin and stigmasterol, ensuring effective inflammation reduction while minimizing systemic exposure.

The thermal instability of stigmasterol aligns with the findings of Bai et al. [32], who reported that the rate of chemical conversion and degradation of stigmasterol increases with elevated temperatures. β-amyrin, on the other hand, had more thermal stability than stigmasterol. The heat sensitivity of β-amyrin was confirmed by da Silva Júnior reporting that β-amyrin had zero-kinetic degradation upon increased temperature [33]. The higher temperature likely induced faster chemical breakdown of both compounds. Overall, the results indicate that the formulation is more stable at 4 °C and experiences significant degradation at elevated temperatures, particularly for stigmasterol.

The degradation kinetics study demonstrated that the remaining percentages of β-amyrin and stigmasterol in the YTPS-FFS formulation stored at 4 °C for 0, 1, 3, and 6 months were significantly higher than those observed at storage temperatures of 30 °C and 40 °C. The zero-order kinetics observed suggest that β-amyrin degradation occurred at a constant rate, irrespective of its concentration in the formulation. The same kinetic degradation behavior of β-amyrin was similar to da Silva Júnior et al. [33]. These results indicate that while higher temperatures accelerate degradation, β-amyrin remains relatively stable within the YTPS-FFS formulation, particularly under cooler storage conditions. The observed reduction in the concentrations of β-amyrin and stigmasterol at elevated temperatures (30 °C and 40 °C) can be attributed to their sensitivity to heat, which accelerates degradation processes. Stigmasterol exhibited a higher rate of degradation compared to β-amyrin under these conditions, suggesting it is more thermally labile. The %remaining of stigmasterol indicates a progressive increase in degradation rate with higher storage temperatures, suggesting that thermal conditions significantly influence the degradation mechanism [24,34]. The results also suggest that stigmasterol’s structural characteristics, particularly its double bonds and hydroxyl group, may make it more prone to oxidation and other degradation pathways [35,36,37]. The primary oxidation products of stigmasterol include a variety of oxidized derivatives that result from structural modifications induced by oxidative stress. Among the major oxidation products are 7-ketosterols, 7-hydroxysterols, triols, 5,6-epoxysterols, and 25-hydroxysterols. These compounds are formed through oxidative processes that target specific sites on the sterol molecule, particularly the double bond in the steroid nucleus and the hydroxyl group at C3 [38]. This indicates that lower temperatures are more favorable for maintaining the stability of these bioactive compounds over time.

The differential stability of these compounds underscores the importance of temperature as a critical factor influencing the longevity and efficacy of the YTPS-FFS formulation. Elevated temperatures likely promote mechanisms such as oxidation or hydrolysis, which can compromise the structural integrity of stigmasterol and, to a lesser extent, β-amyrin. These findings highlight the necessity of temperature-controlled storage to minimize degradation, preserve the therapeutic properties of the formulation, and ensure its effectiveness for long-term use. Further investigations into the degradation pathways and the potential use of stabilizing agents could provide additional strategies to enhance the thermal stability of these compounds in the YTPS-FFS formulations.

β-Amyrin was considered thermally stable up to approximately 200 °C, beyond which degradation occurs [34]. Stigmasterol, on the other hand, was safe in the present practices of use and concentration described in the safety assessment by The Cosmetic Ingredient Review (CIR) expert panel [39]. However, comprehensive studies specifically addressing the skin toxicity of β-amyrin are limited. Further research is necessary to conclusively determine its safety profile concerning dermal applications.

## 3. Conclusions

This study demonstrated that the optimized formulation is suitable for a FFS. The formulation holds significant potential for alleviating pain, enhancing patient compliance, and improving drug delivery efficiency. Based on the comprehensive analysis, the formulation containing 10.0% *w*/*w* YTPS ethanolic extract, 10.0% *w*/*w* PVP K-30 as a film-forming agent, and 2% *w*/*w* PG as a plasticizer represented a promising approach for transdermal delivery. Furthermore, the stability studies revealed the importance of temperature-controlled storage to maintain the integrity of bioactive compounds, β-amyrin and stigmasterol, ensuring long-term efficacy. The implemented evaluation framework effectively demonstrated the formulation’s strong performance across six key criteria: pH, spray angle, drying time, stickiness, washability, and bioactive compound stability, highlighting its suitability for practical applications. These parameters proved instrumental in identifying an optimal formulation that balances functionality and stability, underscoring the importance of systematic assessment in dosage form development. The findings highlight the potential of the YTPS-FFS as an effective, standardized, and patient-friendly option for modernized traditional medicine in the treatment of musculoskeletal pain and inflammation.

## 4. Materials and Methods

### 4.1. Materials

β-Amyrin and stigmasterol reference standards were purchased from ChemFaces (Wuhan, China). Absolute ethanol (analytical grade), acetonitrile (HPLC grade), methanol (HPLC grade), and water (HPLC grade) were bought from Daejung Chemical Co. (Busan, Republic of Korea). PVP K30 (food grade), propylene glycol (food grade), and ethanol (food grade) were bought from Bangkok Chemical Co. (Bangkok, Thailand).

### 4.2. Methods

#### 4.2.1. Plant Material and Extraction Procedure

All plants listed in Table 7 were collected from their respective localities in Thailand and identified by Mr. Nopparut Toolmal, a plant taxonomist. However, *Ferula assa-foetida*, *Aloe vera*, and *Aloe ferox* were acquired from a Thai traditional drug store. Voucher specimens of these plants were deposited at the Thai Traditional Medicine Herbarium (TTM) in Bangkok, Thailand. The specific parts of each herbal component used in the YTPS formulary were referenced from the National Thai Traditional Medicine Formulary, published by the Ministry of Public Health, Thailand. This information was reviewed and validated by an expert committee consisting of an ancient linguist, a Thai traditional medicine practitioner, a botanist, a pharmacist, and a medical doctor.

#### 4.2.2. Preparation of Plant Extracts

To ensure the correct plant parts were used, all foreign materials were removed from the collected specimens. Each herbal component of the YTPS formulation was thoroughly cleaned, cut, and dried at 40 °C using a hot air oven (Binder^®^, BINDER GmbH, Tuttlingen, Germany) overnight until fully desiccated. The dried plant materials were then milled, sieved, and proportionally weighed as outlined in Table 7 followed by dry mixing. A total of 300 g of the YTPS mixed powder, along with the individual plant ingredients, were macerated in 3000 mL of 95% ethanol and periodically agitated using an orbital shaker (Hysc lab^®^, Hanyang Science Lab Co., Ltd., Seoul, Republic of Korea) for 48 h. The macerated samples were filtered through filter paper, and the filtrate was evaporated in a vacuum oven at 40 °C under a pressure of 300 millibars until dry. The dried extracts were then stored at 2 °C until further preparation of the FFS.

#### 4.2.3. Pre-Formulation of YTPS-FFS

The pre-formulation of the YTPS-FFS was studied to determine the most optimized FFS base formulation suitable for the YTPS extract, as shown in Table 8.

#### 4.2.4. Preparation of YTPS-FFS

The YTPS-FFS solution was prepared by gradually dispersing 10% *w/v* of PVP K-30 into a 100 mL beaker containing 40 mL of 95% ethanol. The mixture was continuously stirred using a magnetic stirrer (GUARDIAN™ 3000, OHAUS, Parsippany, NJ, USA) until the film-forming agent was fully dissolved. Subsequently, 10% *w/v* of the YTPS ethanolic extract and 2% *v/v* of propylene glycol were added to the solution, with continuous stirring to ensure complete dissolution of the extract. Finally, purified water was added to the mixture to bring the total volume to 50 mL. This systematic approach ensured the thorough dissolution and uniform distribution of all components [40].

#### 4.2.5. Physical Characteristic and Stability Evaluation

Physical evaluation of the YTPS-FFS was conducted according to Umar et al. [9,41] and Bakhrushina et al. [15]^.^ The appearance, density, pH value, stickiness, water washability, evaporation time, spray angle, weight delivered after every actuation, weight loss, and theoretical film thickness of the YTPS-FFS were evaluated. The viscosity of the YTPS-FFS was evaluated by using a rheometer (Brookfield Engineering Laboratories, Inc., Middleboro, MA, USA).

##### Appearance

A visual inspection of the YTPS-FFS was performed to assess the general appearance, homogeneity, and clarity. This inspection included the examination of the formulation for any visible signs of phase separation, foreign particles, precipitation, or discoloration [42].

##### Density

The density of the YTPS-FFS was determined by pipetting 1 mL of the YTPS-FFS solution and weighing the liquid at 25 °C [43]. The density was calculated using the equation below.

(1) $$\mathrm{Density}\;\left(g/{\mathrm{cm}}^{3}\right)=\frac{\mathrm{Mass}\;\left(g\right)}{\mathrm{Volume}\;\left(\mathrm{mL}\right)}$$

##### pH Values

The pH of the YTPS-FFS was determined using a pH meter (pH meter SG23, METTLER-TOLEDO, Greifensee, Switzerland).

##### Stickiness

The stickiness of the YTPS-FFS formulation was assessed by gently pressing cotton wool onto the film formed in a Petri dish. The degree of adhesion of the cotton fibers to the film served as an indicator of its stickiness. The extent of fibers adhering to the film was categorized as high, medium, or low, based on the amount of cotton wool attached. A substantial amount of attached fibers was classified as high stickiness, a moderate amount as medium, and minimal or no fiber attachment as low stickiness [9].

##### Water Washability

The water washability of the YTPS-FFS film was evaluated by rinsing the film formed on human skin with running tap water. The ease with which the film could be removed was assessed using a numerical score. A rating of “1” indicated that the film could be easily removed in less than 1 min. A rating of “2” corresponded to a more time-consuming removal process, requiring between 1 and 2 min. A rating of “3” denoted significant difficulty in removal, taking more than 2 min [9,44]. This scale provided a qualitative measure of the formulation’s ability to be effectively washed off with water.

##### Evaporation Time

The drying time of the YTPS-FFS was determined by measuring the time required for the film to fully dry on a filter paper using a calibrated stopwatch [45]. The full dry state can be confirmed by directly observing the drying process. A film-forming solution was applied to the surface, and to test if the film had dried, a glass plate was gently placed against the film without applying pressure. If no water adheres to the glass plate, the film is considered fully dry [10].

##### Spray Angle

**Figure 4:**
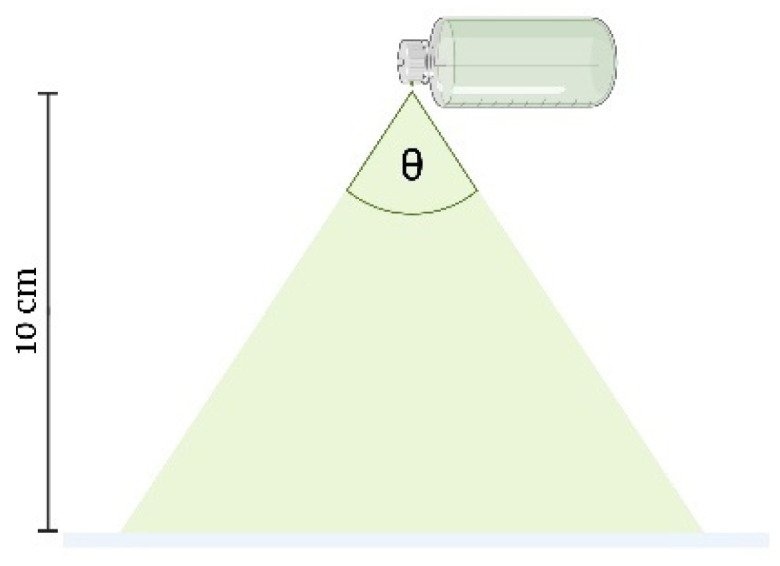
Spray angle measurements (the drawing was adapted from Umar, A. K., et al. (2020) [10]).

The YTPS-FFS was sprayed onto filter paper to measure the radius of the droplets dispensed from the spray bottle. The spray bottle was positioned at a height of 10 cm above the filter paper, which was placed on a flat surface, as shown in Figure 4. Once the YTPS-FFS film had completely dried, the radius of the resulting droplets was measured. The spray angle was calculated according to the following equation [32]:

(2) $$Spray\;angle\;\left(\theta \right)=\left(\frac{l}{r}\right)\;$$

Here, *l* represents the distance between the paper surface and the nozzle, while *r* denotes the radius of the resulting circle.

##### Weight Delivered After Every Actuation

The weight delivered after every actuation was measured by weighing the YTPS-FFS bottle after every actuation. The equation provided below is used to calculate the spray weight delivered after every actuation [9].

(3) $$W_{s}=W_{0}-W_{t}$$

In this equation, *W_s_* represents the spray weight, *W_t_* is the weight of the film-forming solution after spraying, and *W*_0_ is the weight of the solution prior to spraying.

##### Weight Loss

The YTPS-FFS bottles were weighed by an analytical balance (MS204TS/00, METTLER-TOLEDO, Greifensee, Switzerland) to record the weight loss during the stability study. The weight loss of the YTPS-FFS was calculated using the equation below [9].

(4) $$\;W_{l}=W_{0}-W_{t}\;$$

In this equation, *W_l_* represents the spray weight, *W_t_* is the weight of the film-forming solution after spraying, and *W*_0_ is the weight of the solution prior to spraying.

##### Theoretical Film Thickness

The thickness of the film was determined using the equation outlined in the ASTM E0252-06R13 standard [32].

(5) $$\mathrm{Thickness}\;\left(\mathrm{cm}\right)=\frac{\mathrm{Mass}\;\left(g\right)}{\mathrm{Area}\;\left({\mathrm{cm}}^{2}\right)\times \;\mathrm{Density}\;\left(g/{\mathrm{cm}}^{3}\right)}$$

#### 4.2.6. Method Validation of HPLC Analysis

The RP-HPLC method analysis for simultaneous quantification of β-amyrin and stigmasterol in the YTPS-FFS was validated according to the Association of Official Analytical Collaboration (AOAC) guidelines, guidelines for standard method performance requirements 2016 [46], in terms of specificity, linearity, range, accuracy, precision, sensitivity, robustness, and system suitability.

##### Preparation of Standard Solution

β-amyrin and stigmasterol were accurately weighed, dissolved in methanol, and sonicated to prepare stock solutions (1.0 mg/mL each) in 10 mL volumetric flasks. Working standard solutions (10–100 µg/mL) were subsequently prepared by diluting 500 µL of each stock solution, followed by vortex mixing to ensure homogeneity.

##### Preparation of Sample Solution

The YTPS-FFS was diluted with methanol (1:1) and centrifuged. The supernatant was then filtered through a 0.22 µm nylon syringe filter to remove particulates, preparing it for HPLC analysis.

##### Chromatographic Conditions

The Thermo Scientific™ Vanquish™ LC System (Germering, Germany) with a quaternary pump and photodiode array detector was used for method validation. Separation was performed on a Hypersil GOLD™ C_18_ column (250 mm × 4.6 mm i.d.) at 25 °C, with a mobile phase of acetonitrile and methanol (96:5% *v*/*v*) at a flow rate of 1.5 mL/min. Samples (20 µL) were analyzed with UV detection at 202 nm over a 20-min run time using Chromeleon^®^ software (Version 7.3.1).

##### Specificity

The specificity of the analytical method was evaluated by quantifying β-amyrin and stigmasterol in the YTPS-FFS and examining the chromatogram for interfering peaks at their retention times.

##### Linearity and Range

To assess linearity and range, each working standard solution was injected three times, and the peak areas were recorded for each concentration. A calibration curve was established by plotting concentration against peak area for both β-amyrin and stigmasterol.

##### Accuracy

Accuracy was evaluated by calculating the percentage recovery of β-amyrin and stigmasterol spiked into pre-analyzed YTPS-FFS samples at low, middle, and high concentrations. Triplicate analyses were performed at each level to determine recovery, bias, and %RSD, ensuring method precision and reliability.

(6) $$Recovery\;\left(\%\right)=\frac{Amount\;found-Amount\;added}{Amount\;added}\times 100$$

##### Precision

Intra-day precision was assessed by triplicate analysis of spiked samples at three concentration levels within a single day, while inter-day precision was evaluated over three consecutive days. These assessments ensured the method’s repeatability and consistency over short- and long-term intervals.

##### Sensitivity

The limit of detection (LOD) was employed to assess the sensitivity of the analytical method. The limit of quantitation (LOQ) was calculated as the lowest amount that could be quantified.

(7) $$\mathrm{LOD}=\frac{3\mathrm{SD}\;\mathrm{of}\;\mathrm{intercepts}}{\mathrm{Mean}\;\mathrm{of}\;\mathrm{slope}}$$

(8) $$\mathrm{LOQ}=\frac{10\mathrm{SD}\;\mathrm{of}\;\mathrm{intercepts}}{\mathrm{Mean}\;\mathrm{of}\;\mathrm{slope}}$$

##### Robustness

The parameters selected for studying the robustness included variations in flow rate (±0.2 mL/min) and mobile phase ratio (±2% *v*/*v*). The impact of these altered parameters on the analysis of β-amyrin and stigmasterol in the YTPS-FFS was assessed based on %recovery and %RSD results.

##### System Suitability

The test was conducted by performing six replicate injections of a mixed standard solution containing 60 µg/mL of β-amyrin and stigmasterol. Subsequently, system suitability parameters were evaluated, which included retention time, resolution, theoretical plate count, separation factor, retention factor, asymmetry, and tailing factor.

#### 4.2.7. Kinetic Degradation of β-Amyrin and Stigmasterol in the YTPS-FFS During Storage

The active compounds of the YTPS-FFS, β-amyrin and stigmasterol, were determined by mixing the YTPS-FFS solution with ethanol prior to an HPLC analysis.

The degradation kinetics of β-amyrin and stigmasterol were analyzed by fitting the experimental data to various kinetic models, including zero-order, first-order, and second-order equations. The kinetics Equations (9)–(11) used are described below [47].

Zero-order kinetics:

(9) $$C_{t}=C_{0}+k_{0}t$$

Here, *C_t_* denotes the concentration of active compounds at time, *C*_0_ represents the initial concentration of active compounds, and k_0_ is the rate constant for zero-order kinetics.

First-order kinetics:

(10) $$\ln\;C_{t}=\ln\;C_{0}+k_{1}t$$

In this equation, *C_t_* represents the concentration of active compounds at time, *C*_0_ represents the initial concentration of active compounds, and *k*_1_ is the rate constant for first-order kinetics.

Second-order kinetics:

(11) $$\frac{1}{\;C_{t}}-\frac{1}{C_{0}}=k_{2}t$$

The *C_t_* represents the concentration of the active compound at a given time *t*, *C*_0_ denotes the initial concentration of the active compound, and *k*_2_ is the rate constant associated with second-order kinetics.

#### 4.2.8. Statistical Analysis

The data were gathered and analyzed using GraphPad Prism 9 (La Jolla, CA, USA) with one-way analysis of variance (ANOVA) and Dunnett multiple comparison test as a post hoc analysis to assess the significance of differences among the groups. A *p*-value of less than 0.05 was deemed statistically significant (Supplementary Tables S1–S7).

## Figures and Tables

**Table 1:** Physical properties of the FFS base.

Formulation Number	Physical Properties				
	Appearance	Viscosity	Spray Ability	Drying Time	Film Characteristic
1	Clear	Medium-High	Poor	N/A	No film was formed
2	Turbid	High	N/A	N/A	N/A
3	White–turbid	High	N/A	N/A	N/A
4	Clear	Low	Good	<1 min	No film was formed
5	Clear	Low	Good	<1 min	No film was formed
6	Clear	Low	Good	<1 min	No film was formed
7	Clear	Low	Good	1–2 min	The film was not completely formed
8	Clear	Low	Good	1–2 min	The film was not completely formed
9	Clear	Low	Good	>2 min	The film was completely formed
10	Clear	Low	Good	1–2 min	White, turbid film was formed

Results are collated from a sample size of three (*n* = 3) per experiment. Drying rate definition: high (<1 min), medium (1–2 min), and slow (>2 min).

**Table 2:** Physical stability of the YTPS-FFS formulation.

Physical Properties	Time	Temperature		
		4 °C	30 °C	40 °C
Appearance	0 month	Clear green solution	Clear green solution	Clear green solution
	1 month	Clear green solution	Clear green solution	Clear green solution
	3 months	Clear green solution	Clear green solution	Clear green solution
	6 months	Clear green solution	Clear green solution	Clear green solution
pH	Initial	4.88 ± 0.00	4.93 ± 0.00	4.72 ± 0.00
	1 month	4.95 ± 0.00	4.92 ± 0.00	4.89 ± 0.00
	3 months	4.88 ± 0.00	4.87 ± 0.00	4.87 ± 0.00
	6 months	4.89± 0.00	4.79± 0.00	4.73± 0.00
Density (g/cm^3^)	0 month	0.8896 ± 0.0100	0.8831 ± 0.0000	0.8885 ± 0.0200
	1 month	0.8450 ± 0.0000 **	0.8403 ± 0.0000 ***	0.8383 ± 0.0000 ***
	3 months	0.9086 ± 0.0000	0.9117 ± 0.0000 *	0.9069 ± 0.0000
	6 months	0.8471 ± 0.0000 **	0.8475 ± 0.0000 **	0.8397 ± 0.0000 ***
Weight (g)	0 month	55.04 ± 0.24	54.99 ± 0.78	55.89 ± 0.52
	1 month	55.02 ± 0.23	54.96 ± 0.79	55.45 ± 0.51
	3 months	54.97 ± 0.23	54.91 ± 0.77	54.84 ± 0.56
	6 months	54.95 ± 0.24	54.88 ± 0.78	54.45 ± 0.57
Spray angle (°)	0 month	58.41 ± 0.17	58.69 ± 0.20	60.10 ± 0.18
	1 month	59.18 ± 0.53	58.62 ± 0.17	59.96 ± 0.36
	3 months	58.48 ± 0.43	58.27 ± 0.10	58.34 ± 0.20 ***
	6 months	58.48 ± 0.52	57.65 ± 0.39**	55.58 ± 0.29 ****
Weight delivered after actuation (g)	0 month	0.13 ± 0.00	0.14 ± 0.00	0.12 ± 0.00
	1 month	0.1063 ± 0.0000 *	0.1280 ± 0.0000 ***	0.1327 ± 0.0000 **
	3 months	0.1086 ± 0.0000 *	0.1291 ± 0.0000 ***	0.1333 ± 0.0000 **
	6 months	0.1193 ± 0.0100	0.1279 ± 0.0000 ***	0.0957 ± 0.000 ****
Evaporation time (min)	0 month	1.18 ± 0.06	1.22 ± 0.03	1.11 ± 0.02
	1 month	1.05 ± 0.02 *	1.27 ± 0.02	1.17 ± 0.03
	3 months	1.15 ± 0.03	1.20 ± 0.02	1.04 ± 0.02
	6 months	1.13 ± 0.03	1.14 ± 0.01 *	1.17 ± 0.03
Film appearance	0 month	Flexible, no rupture	Flexible, no rupture	Flexible, no rupture
	1 month	Flexible, no rupture	Flexible, no rupture	Flexible, no rupture
	3 months	Flexible, no rupture	Flexible, no rupture	Flexible, no rupture
	6 months	Flexible, no rupture	Flexible, no rupture	Flexible, no rupture
Stickiness	0 month	Low	Low	Low
	1 month	Low	Low	Low
	3 months	Low	Low	Low
	6 months	Low	Low	Low
Washability	0 month	1	1	1
	1 month	1	1	1
	3 months	1	1	1
	6 months	1	1	1

Results are expressed as the mean ± standard deviation (SD), with a sample size of three (*n* = 3) per experiment. The data were analyzed by GraphPad Prism version 9.5.0 with one-way ANOVA and Dunnett’s multiple comparisons test (statistical significance denoted by * *p* < 0.05, ** *p* < 0.01, *** *p* < 0.001, and **** *p* < 0.0001 compared with the initial value).

**Table 3:** Accuracy of the validated analytical method.

Compounds	Pre-Analyzed Sample (µg/mL)	Amount of Standard Added (µg/mL)	Theoretical Content (µg/mL)	Recorded Amount ±SD (µg/mL)	Average %Recovery	%RSD
β-Amyrin	22.37	25	47.37	47.19 ± 0.55	99.63	1.17
		50	72.37	72.65 ± 0.47	100.40	0.65
		75	97.37	97.26 ± 0.90	99.89	0.92
Stigmasterol	7.43	25	32.43	32.26 ± 0.35	99.48	1.07
		50	57.43	57.68 ± 0.38	100.44	0.66
		75	82.43	82.35 ± 0.92	99.90	1.12

Results are expressed as the mean ± SD based on analysis of triplicate samples (*n* = 3).

**Table 4:** Precision of the validated analytical method.

Compounds	Amount of Standard Added (µg/mL)	Intra-Day Precision		Inter-Day Precision	
		Average % Recovery	%RSD	Average % Recovery	%RSD
β-Amyrin	25	98.41	0.53	98.81	0.72
	50	99.41	0.22	99.73	0.58
	75	100.67	0.09	100.31	0.40
Stigmasterol	25	99.37	0.58	99.58	0.28
	50	98.80	0.13	99.84	0.91
	75	99.97	0.54	99.90	0.06

Results are expressed as the mean ± SD based on analysis of triplicate samples (*n* = 3).

**Table 5:** Robustness of the validated analytical method.

Changes in Condition	Compounds	Amount of Standard Added (µg/mL)	% Recovery	
			Average	%RSD
**Flow rate**				
1.3 mL/min	β-Amyrin	25	99.04	1.63
		50	100.22	0.48
		75	99.28	0.12
	Stigmasterol	25	99.43	0.26
		50	101.34	0.25
		75	100.38	0.10
1.7 mL/min	β-Amyrin	25	98.87	0.52
		50	99.16	0.75
		75	100.38	0.28
	Stigmasterol	25	99.05	0.48
		50	99.82	0.04
		75	100.11	0.04
**Mobile phase ratio**				
ACN:MeOH (94:6)	β-Amyrin	25	100.91	1.36
		50	101.14	0.76
		75	98.78	1.72
	Stigmasterol	25	100.60	0.49
		50	100.25	0.30
		75	99.63	0.54
ACN:MeOH (98:2)	β-Amyrin	25	99.17	1.26
		50	104.04	0.36
		75	96.78	0.48
	Stigmasterol	25	102.36	0.39
		50	99.92	0.87
		75	99.26	1.07

Results are expressed as the mean ± SD based on analysis of triplicate samples (*n* = 3).

**Table 6:** System suitability parameters of the validated analytical method.

Parameters	β-Amyrin		Stigmasterol	
	Average	%RSD	Average	%RSD
Retention time	9.75 ± 0.36	3.72	10.71 ± 0.50	4.65
Resolution	3.09 ± 0.25	8.24	N/A	N/A
Theoretical plate count	17,589.00 ± 135.76	0.77	17,715.50 ± 852.06	4.81
Separation factor	1.13 ± 0.37	32.87	N/A	N/A
Retention factor	7.61 ± 0.01	0.09	8.57 ± 0.50	5.89
Asymmetry	1.02 ± 0.01	0.70	1.03 ± 0.01	1.37
Tailing factor	1.00 ± 0.00	0.00	1.08 ± 0.03	2.91

Results are expressed as the mean ± SD based on analysis of triplicate samples (*n* = 3).

**Table 7:** YTPS herbal composition.

No.	Plant Scientific Name	Parts Used	%*w*/*w*	Voucher Specimen
1	*Putranjiva roxburghii* Wall.	Leaves	37	TTM0005479
2	*Senna siamea* (Lam.) H.S. Irwin and Barneby	Leaves	9	TTM0005489
3	*Baliospermum solanifolium* (Burm.) Suresh	Leaves	9	TTM0005473
4	*Cymbopogon nardus* (L.) Rendle	Rhizomes and leaves	9	TTM0005477
5	*Tamarindus indica* L.	Leaves	9	TTM0005469
6	*Melia azedarach* L.	Leaves	9	TTM0005488
7	*Boesenbergia rotunda* (L.) Mansf.	Rhizomes and roots	2	TTM0005476
8	*Allium sativum* L.	Bulbs	2	TTM0005474
9	*Alpinia galanga* (L.) Willd.	Rhizomes	2	TTM0005475
10	*Piper nigrum* L.	Fruits	2	TTM0005490
11	*Ferula assa-foetida* L.	Resin from roots	2	TTM1000743
12	*Aloe vera* (L.) Burm.f.	Resin	2	TTM1000744
13	*Allium ascalonicum* L.	Bulbs	2	TTM0005478

**Table 8:** The FFS base optimization.

Excipients	Function	Formulation Number (% *w*/*v*)									
		1	2	3	4	5	6	7	8	9	10
Hydroxypropyl methylcellulose (HPMC)	film-forming agent	1	3	5	-	-	-	-	-	-	-
Polyvinylpyrrolidone K30 (PVP-K30)	film-forming agent	-	-	-	1	2	3	4	5	10	-
Eudragit^®^	film-forming agent	-	-	-	-	-	-	-	-	-	10
Propylene glycol (PG)	plasticizer	2	2	2	2	2	2	2	2	2	0.5
Purified water	solvent	q.s.	q.s.	q.s.	q.s.	q.s.	q.s.	q.s.	q.s.	q.s.	-
95% ethanol	solvent	70	70	70	70	70	70	80	80	80	100

## Data Availability

Data are contained within the article and Supplementary Materials.

## Supplementary Materials

The following supporting information can be downloaded at: https://www.mdpi.com/article/10.3390/gels11010064/s1. Figure S1. Finished product macroscopic morphology (A) In a spray bottle (B) the dried film on the petri dish with black background (C) the dried film on the petri dish with white background. Figure S2. (A) β-amyrin and (B) stigmasterol standard calibration curve. Table S1. Results of Dunnett’s multiple comparisons test for β-amyrin concentration. Table S2. Results of Dunnett’s multiple comparisons test for stigmasterol concentration. Table S3. Results of Dunnett’s multiple comparisons test for density. Table S4. Results of Dunnett’s multiple comparisons test for evaporation time. Table S5. Results of Dunnett’s multiple comparisons test for film thickness. Table S6. Results of Dunnett’s multiple comparisons test for spray angle. Table S7. Results of Dunnett’s multiple comparisons test for weight delivered after actuation.
