# Supplementary material for: Development, Optimization, and Stability Study of a Yataprasen Film-Forming Spray for Musculoskeletal Pain Management
Source: Gels. 2025 Jan 15;11(1):64. doi: 10.3390/gels11010064 (PMC11764686; doi:10.3390/gels11010064)
Supplement: Supplementary file 1 [file gels-11-00064-s001.zip › gels-3392186-supplementary.pdf]

Supplementary data

Methods

1. Kinetic Degradation of β-amyrin and Stigmasterol in the YTPS-FFS During Storage

The active compounds of the YTPS-FFS, β-amyrin, and stigmasterol, were determined by mixing the YTPS-FFS solution with ethanol in a 1:1 ratio. The mixture was then thoroughly mixed using a vortex mixer (Vortex-Genie® 2, Scientific Industries, Bohemia, New York, USA), followed by centrifugation at 15,000 rpm for 5 mins (Mikro 220R, Hettich, Germany), filtered through a 0.22 μM Nylon syringe filter prior an HPLC analysis.

The degradation kinetics of β-amyrin and stigmasterol were analyzed by fitting the experimental data to various kinetic models, including zero-order, first-order, and second-order equations. The most appropriate kinetic model describing the degradation process was determined by comparing the correlation coefficient (r^2) values obtained for each model. The model with the highest (r^2) value was considered the best fit, providing insight into the reaction mechanism and the dependence of lutein degradation on time and other influencing factors. The kinetics equations (1)-(3) used are described below [1].

Zero-order kinetics

$$C_t = C_0 + k_0 t \tag{1}$$

Here, C_t denotes the concentration of active compounds at time, C_0 represents the initial concentration of active compounds, and k_0 is the rate constant for zero-order kinetics.

First-order kinetics

$$ln\ C_t = ln\ C_0 + kt \tag{2}$$

In this equation, C_t represents the concentration of active compounds at time, C_0 represents the initial concentration of active compounds, and k is the rate constant for first-order kinetics.

Second-order kinetics

$$\frac{1}{[c]} - \frac{1}{[C]_0} = kt \tag{3}$$

The C_t represents the concentration of the active compound at a given time t, C_0 denotes the initial concentration of the active compound, and k is the rate constant associated with second-order kinetics.

2. Method Validation of HPLC Analysis

The RP-HPLC method analysis for simultaneous quantification of β-amyrin and stigmasterol in the YTPS-FFS was validated according to the association of official analytical collaboration (AOAC) guidelines, guidelines for standard method performance requirements 2016 [2], in terms of specificity, linearity, range, accuracy, precision, sensitivity, robustness, and system suitability.

Preparation of Standard Solution

β-amyrin and stigmasterol were accurately weighed and transferred into a 10 mL volumetric flask. Following the addition of 5 mL of methanol, the flask was sonicated until the standards were completely dissolved. The volume was made up to 10 mL with methanol, resulting in a stock solution with a concentration of 1.0 mg/mL for each compound. From these stock solutions, working standard solutions were prepared by transferring 500 μL of each standard into a 1.5 mL centrifuge tube. The mixture was thoroughly mixed with a vortex mixer prior to further dilution, yielding final concentrations of 10, 20, 40, 60, 80, and 100 μg/mL for both β-amyrin and stigmasterol.

Preparation of Sample Solution

The YTPS-FFS was diluted with methanol at a 1:1 ratio. The solution was thoroughly mixed using a vortex mixer, followed by centrifugation at 15,000 rpm for 5 min. After centrifugation, the sample solution

was filtered through a 0.22 μM Nylon syringe filter to ensure the removal of particulates, preparing the sample for subsequent HPLC analysis.

Chromatographic Conditions

The Thermo Scientific™ Vanquish™ LC Systems (Germering, Germany) was used for the method validation, consisting of a quaternary pump with a photodiode array detector. The mobile phase was composed of a mixture of acetonitrile and methanol in a ratio of 96:5% V/V. For each analysis, 20 microliters of the standard and sample solutions were injected. The flow rate of the mobile phase was set to 1.5 mL/min, and separation was achieved using a Hypersil GOLD™ C18 column (250 mm × 4.6 mm i.d.) (). The column temperature was maintained at 25 °C throughout the experiment, with UV detection conducted at a wavelength of 202 nm. The total run time for each analysis was set to 20 min. The system was operated using Chromeleon® software (Version 7.3.1). Specificity

To evaluate the specificity of the analytical method, β-amyrin and stigmasterol were quantified within the YTPS-FFS. The chromatogram was carefully examined for any interfering peaks at the retention times corresponding to the analytes. Linearity and Range

To assess linearity and range, each working standard solution was injected three times, and the peak areas were recorded for each concentration. A calibration curve was established by plotting concentration against peak area for both β-amyrin and stigmasterol.

Accuracy

Accuracy was assessed by calculating the percentage recovery of β-amyrin and stigmasterol added to the pre-analyzed YTPS-FFS samples at low, middle, and high concentrations. Each concentration level was prepared in triplicate, and the results were used to calculate the percentage recovery, percentage bias, and relative standard deviation (% RSD), ensuring the method's precision and reliability. The equation below was used to calculate %recovery.

$$Recovery\ (\%) = \frac{Amount\ found - Amount\ added}{Amount\ added} \times 100 \qquad (4)$$

Precision

The intra-day precision of the proposed analytical method was assessed by analyzing the freshly prepared spiked sample solutions in triplicate at three different concentration levels within the same day. To evaluate inter-day precision, the freshly prepared spiked sample solutions were tested in triplicate at the same concentration levels across three consecutive days. These assessments provided a robust evaluation of the method's repeatability and consistency over both short- and long-term intervals.

Sensitivity

The limit of detection (LOD) was employed to assess the sensitivity of the analytical method. The limit of quantitation (LOQ) was calculated as the lowest amount that could be quantified.

$$LOD = \frac{3SD\ of\ intercepts}{Mean\ of\ slope} \qquad (5)$$

$$LOQ = \frac{10SD\ of\ intercepts}{Mean\ of\ slope} \qquad (6)$$

Robustness

The parameters selected for studying the robustness included variations in flow rate (±0.2 mL/min), and mobile phase ration (±2% v/v). The impact of these altered parameters on the analysis of β-amyrin and stigmasterol in the YTPS-FFS was assessed based on %recovery and %RSD results.

System Suitability

The test was conducted by performing six replicate injections of a mixed standard solution containing 60 μg/mL of β-amyrin and stigmasterol. Subsequently, system suitability parameters were evaluated, which included retention time, resolution, theoretical plate count, separation factor, retention factor, asymmetry, and tailing factor.

Results

1. Finished product macroscopic morphology

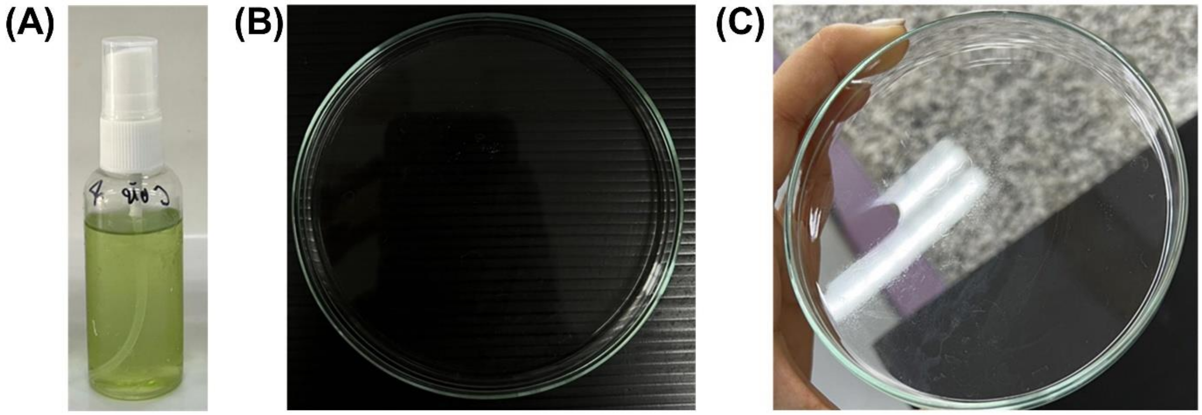

Supplementary Figure S1 Finished product macroscopic morphology (A) In a spray bottle (B) the dried film on the petri dish with black background (C) the dried film on the petri dish with white background

2. Linear regression of β-amyrin and stigmasterol

The standard calibration curve of gallic acid and quercetin are depicted in **Figure 1**.

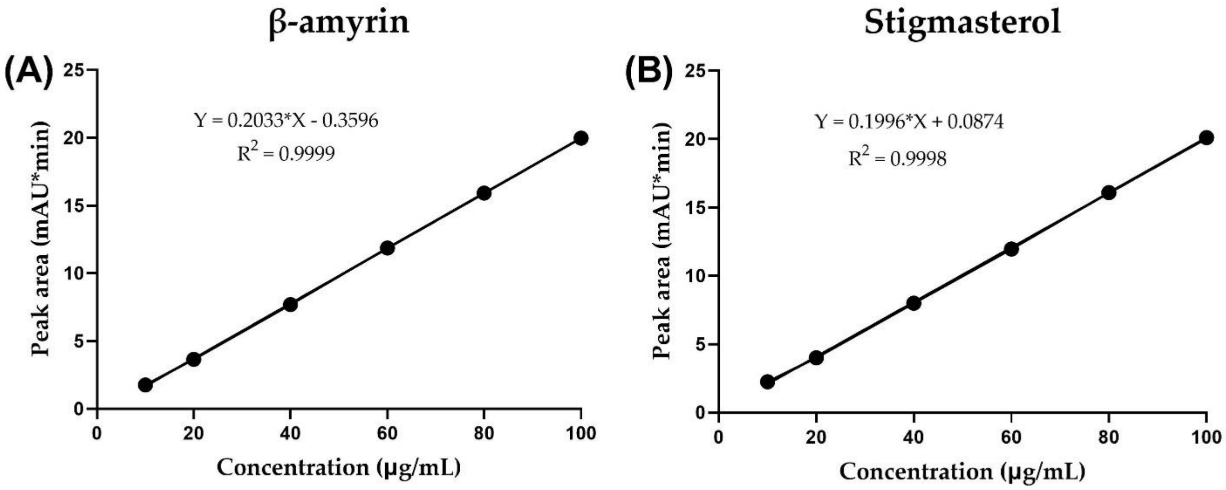

Supplementary Figure S2 (A) β-amyrin and (B) stigmasterol standard calibration curve

3. The statistical analysis of the stability study of the YTPS-FFS formulation

Supplementary Table S1 Results of Dunnett's multiple comparisons test for β-amyrin concentration

Dunnett's multiple comparisons test	Mean Diff.	95.00% CI of diff.	Significant?	Summary	Adjusted P Value
β-amyrin 4 °C					
0 vs. 1	1.11	-1.566 to 3.786	No	ns	0.5351
0 vs. 3	1.147	-1.530 to 3.823	No	ns	0.5122
0 vs. 6	1.01	-1.493 to 3.513	No	ns	0.5544
β-amyrin 30 °C					

0 vs. 1	0.9333	-1.945 to 3.811	No	ns	0.6867
0 vs. 3	0.965	-2.253 to 4.183	No	ns	0.7305
0 vs. 6	2.328	-0.3646 to 5.020	No	ns	0.0893
β-amyrin 40 °C					
0 vs. 1	1.17	-3.244 to 5.584	No	ns	0.7971
0 vs. 3	1.683	-2.731 to 6.097	No	ns	0.5928
0 vs. 6	4.375	0.2460 to 8.504	Yes	*	0.0385

Supplementary Table S2 Results of Dunnett's multiple comparisons test for stigmasterol concentration

Dunnett's multiple comparisons test	Mean Diff.	95.00% CI of diff.	Significant?	Summary	Adjusted P Value
Stigmasterol 4 °C					
0 vs. 1	1.17	-0.3830 to 2.723	No	ns	0.1466
0 vs. 3	2.34	0.7870 to 3.893	Yes	**	0.0057
0 vs. 6	4.278	2.825 to 5.730	Yes	****	<0.0001
Stigmasterol 30 °C					
0 vs. 1	2.34	0.5797 to 4.100	Yes	*	0.0137
0 vs. 3	4.075	2.107 to 6.043	Yes	**	0.0012
0 vs. 6	5.653	3.893 to 7.414	Yes	****	<0.0001
Stigmasterol 40 °C					
0 vs. 1	1.43	-0.9990 to 3.859	No	ns	0.2876
0 vs. 3	4.473	2.044 to 6.902	Yes	**	0.0015
0 vs. 6	6.23	3.958 to 8.502	Yes	****	<0.0001

Supplementary Table S3 Results of Dunnett's multiple comparisons test for density

Dunnett's multiple comparisons test	Mean Diff.	95.00% CI of diff.	Significant?	Summary	Adjusted P Value
Density 4 °C					
0 vs. 1	0.0446	0.02158 to 0.06762	Yes	**	0.0014
0 vs. 3	-0.01903	-0.04205 to 0.003983	No	ns	0.1048
0 vs. 6	0.04253	0.01952 to 0.06555	Yes	**	0.0018
Density 30 °C					
0 vs. 1	0.04927	0.02731 to 0.07122	Yes	***	0.0005

0 vs. 3	-0.02207	-0.04402 to -0.0001091	Yes	*	0.0489
0 vs. 6	0.04213	0.02018 to 0.06409	Yes	**	0.0014
Density 40 °C					
0 vs. 1	0.05127	0.02936 to 0.07317	Yes	***	0.0004
0 vs. 3	-0.0173	-0.03920 to 0.004605	No	ns	0.1226
0 vs. 6	0.04993	0.02803 to 0.07184	Yes	***	0.0005

Supplementary Table S4 Results of Dunnett's multiple comparisons test for evaporation time

Dunnett's multiple comparisons test	Mean Diff.	95.00% CI of diff.	Significant?	Summary	Adjusted P Value
Evaporation time 4 °C					
0 vs. 1	0.1333	0.02368 to 0.2430	Yes	*	0.0202
0 vs. 3	0.03	-0.07966 to 0.1397	No	ns	0.7754
0 vs. 6	0.05	-0.05966 to 0.1597	No	ns	0.4575
Evaporation time 30 °C					
0 vs. 1	-0.05	-0.1133 to 0.01331	No	ns	0.1227
0 vs. 3	0.01333	-0.04998 to 0.07664	No	ns	0.8754
0 vs. 6	0.07667	0.01336 to 0.1400	Yes	*	0.0206
Evaporation time 40 °C					
0 vs. 1	-0.06	-0.1392 to 0.01916	No	ns	0.1403
0 vs. 3	0.06667	-0.01249 to 0.1458	No	ns	0.0982
0 vs. 6	-0.06	-0.1392 to 0.01916	No	ns	0.1403

Supplementary Table S5 Results of Dunnett's multiple comparisons test for film thickness

Dunnett's multiple comparisons test	Mean Diff.	95.00% CI of diff.	Significant?	Summary	Adjusted P Value
Film thickness 4 °C					
0 vs. 1	0.7833	-1.321 to 2.888	No	Ns	0.5991
0 vs. 3	1.92	-0.1847 to 4.025	No	Ns	0.0728
0 vs. 6	0.15	-1.955 to 2.255	No	ns	0.9935
Film thickness 30 °C					

0 vs. 1	0.94	0.03193 to 1.848	Yes	*	0.0431
0 vs. 3	2.18	1.272 to 3.088	Yes	***	0.0003
0 vs. 6	1.993	1.085 to 2.901	Yes	***	0.0006
Film thickness 40 °C					
0 vs. 1	-1.77	-2.765 to -0.7749	Yes	**	0.0023
0 vs. 3	1	0.004927 to 1.995	Yes	*	0.0489
0 vs. 6	5.57	4.575 to 6.565	Yes	****	<0.0001

Supplementary Table S6 Results of Dunnett's multiple comparisons test for spray angle

Dunnett's multiple comparisons test	Mean Diff.	95.00% CI of diff.	Significant?	Summary	Adjusted P Value
Spray angle 4 °C					
0 vs. 1	-0.7733	-2.032 to 0.4856	No	ns	0.2532
0 vs. 3	-0.07	-1.329 to 1.189	No	ns	0.9969
0 vs. 6	-0.07333	-1.332 to 1.186	No	ns	0.9965
Spray angle 30 °C					
0 vs. 1	0.07	-0.6173 to 0.7573	No	ns	0.9819
0 vs. 3	0.42	-0.2673 to 1.107	No	ns	0.2564
0 vs. 6	1.037	0.3494 to 1.724	Yes	**	0.0063
Spray angle 40 °C					
0 vs. 1	0.1433	-0.6301 to 0.9167	No	ns	0.909
0 vs. 3	1.763	0.9899 to 2.537	Yes	***	0.0005
0 vs. 6	4.53	3.757 to 5.303	Yes	****	<0.0001

Supplementary Table S7 Results of Dunnett's multiple comparisons test for weight delivered after actuation

Dunnett's multiple comparisons test	Mean Diff.	95.00% CI of diff.	Significant?	Summary	Adjusted P Value
Weight delivered after actuation 4 °C					
0 vs. 1	0.02	0.001263 to 0.03874	Yes	*	0.037
0 vs. 3	0.02	0.001263 to 0.03874	Yes	*	0.037
0 vs. 6	0.01333	-0.005404 to 0.03207	No	Ns	0.1825

Weight delivered after actuation 30 °C					
0 vs. 1	0.01667	0.009585 to 0.02375	Yes	***	0.0003
0 vs. 3	0.01667	0.009585 to 0.02375	Yes	***	0.0003
0 vs. 6	0.01667	0.009585 to 0.02375	Yes	***	0.0003
Weight delivered after actuation 40 °C					
0 vs. 1	-0.01	-0.01708 to -0.002918	Yes	**	0.0085
0 vs. 3	-0.01	-0.01708 to -0.002918	Yes	**	0.0085
0 vs. 6	0.02333	0.01625 to 0.03042	Yes	****	<0.0001

[1] Bell, L.N. Moisture Effects on Food's Chemical Stability. In *Water Activity in Foods*; 2020; pp. 227-253.

[2] AOAC International. *Guidelines for Standard Method Performance Requirements*; AOAC Official Methods of Analysis, 20th ed.; AOAC International: Rockville, MD, USA, 2016.
